# KRT18 Modulates Alternative Splicing of Genes Involved in Proliferation and Apoptosis Processes in Both Gastric Cancer Cells and Clinical Samples

**DOI:** 10.3389/fgene.2021.635429

**Published:** 2021-07-05

**Authors:** Biao Chen, Ximing Xu, Dan-dan Lin, Xin Chen, Yang-tao Xu, Xin Liu, Wei-guo Dong

**Affiliations:** ^1^Cancer Center, Renmin Hospital of Wuhan University, Wuhan, China; ^2^Department of Gastroenterology, Renmin Hospital of Wuhan University, Wuhan, China

**Keywords:** KRT18, RNA-Seq, alternative splicing, cell cycle and apoptosis, gastric cancer

## Abstract

Keratin 18 (KRT18), one of the most abundant keratins in epithelial and endothelial cells, has been reported to be aberrantly expressed in many malignancies and extensively regarded as a biomarker and important regulator in multiple cancers, including gastric cancer (GC). But the molecular regulatory mechanisms of KRT18 in GC patients and cells are largely unknown. In the present study, we analyzed the expression level of KRT18 in 450 stomach adenocarcinoma tissue samples from TCGA database and found a significantly higher expression level in tumor tissues. We then explored the potential functions of KRT18 in AGS cells (human gastric adenocarcinoma cell line) by KRT18 knockdown using siRNA and whole transcriptome RNA-seq analysis. Notably, KRT18 selectively regulates expression of cell proliferation and apoptotic genes. Beyond this, KRT18 affects the alternative splicing of genes enriched in apoptosis, cell cycle, and other cancer-related pathways, which were then validated by reverse transcription–quantitative polymerase chain reaction approach. We validated KRT18-KD promoted apoptosis and inhibited proliferation in AGS cells. We then used RNA-seq data of GC samples to further demonstrate the modulation of KRT18 on alternative splicing regulation. These results together support the conclusion that KRT18 extensively modulates diverse alternative splicing events of genes enriched in proliferation and apoptosis processes. And the dysregulated splicing factors at transcriptional or posttranscriptional level by KRT18 may contribute to the alternative splicing change of many genes, which expands the functional importance of keratins in apoptotic and cell cycle pathways at the posttranscriptional level in GC.

## Background

Gastric cancer (GC) is one of the most common and deadly neoplasms in the world, with the fifth incidence and the third mortality in all cancers around the world, following lung and colorectal cancer (Van Cutsem et al., 2016; Rawla and Barsouk, 2019; Thrift and El-Serag, 2020). The incidence data of GC show strong geographical variation. More than half of the cases were found in developing countries. The high-risk areas includes East Asia, Eastern Europe, and some parts of America (Sitarz et al., 2018). The main risk factors of GC include dietary factors and *Helicobacter pylori* infection. It is found that various salt-preserved foods and low consumption of vegetables and fruits may make the risk of GC higher (Hartgrink et al., 2009). Studies have also revealed that *H. pylori* infection is an important cause of the gastritis before most GCs (Loor and Dumitrascu, 2016; Van Cutsem et al., 2016). *H. pylori* infection was reported to trigger GC by multiple pathogenic mechanisms, including endoplasmic reticulum stress and the unfolded protein response, autophagy, oxidative stress, and inflammation (Amieva and Peek, 2016; Diaz et al., 2018). Genetic cause of GC refers mutations of some genes, including *CDH1*, *ARID1A*, *TP53*, *MUC6*, *CTNNA2*, and others (Wang et al., 2014). It was reported that HER2 positivity is associated with worsening prognosis, increased disease invasiveness, and shortened survival time in approximately 12–20% of GCs (Gravalos and Jimeno, 2008; Van Cutsem et al., 2015). Even though multidisciplinary approach has been gradually applied to treat GC with the advances in diagnosis, genomic classification, surgical resection, chemoradiation, and immune therapies, the prognosis for advanced GC remains unsatisfactory (Sitarz et al., 2018). Therefore, it is very important to identify molecular markers and therapeutic targets to improve the effectiveness of GC treatment.

Keratin 18 (CK18/KRT18) is one of the keratins (cytokeratins), which are intermediate filaments (IFs) essential for tissue completion (Hesse et al., 2001; Coulombe and Wong, 2004). Keratins primarily play a role in protecting epithelial cells from both mechanical and non-mechanical stressors (Dmello et al., 2019). Keratin filaments can be dynamically remodeled, undergoing reorganization upon various mechanical and non-mechanical stimuli to regulate cellular processes, including cell migration and signaling (Chung et al., 2013). It has been reported that keratins have many roles in tumorigenesis (Hu et al., 2017), progression (Sharma et al., 2019), and drug responsiveness processes (Yin et al., 2016; Zhang et al., 2016), which allows them to be regarded as diagnostic markers and prognostic markers in tumors.

The staining of IFs, most notably keratins, has been used to be a useful tool to diagnose human tumors clinically (Mohme et al., 2017; Sharma et al., 2019). KRT18, as one of the most abundant keratins in epithelial and endothelial cells, was reported to be aberrantly expressed in many malignancies and extensively regarded as a diagnostic and prognostic marker in cancers, including non–small cell lung cancer (Zhang et al., 2016), GC (Bilici, 2015; Nagel et al., 2018), hepatocellular cancer (Golob-Schwarzl et al., 2019), breast cancer (Bozza et al., 2018), and colorectal cancer (Zhang et al., 2019a). It is the major component of IFs in epithelial cells and released from cells into the blood in either intact (M65) or caspase-cleaved forms (M30) during apoptotic and necrotic cell death (Kramer et al., 2004; Schutte et al., 2004). Notably, the M30 and M65 have prognostic and predictive significance in advanced GC (Bilici, 2015; Nagel et al., 2018).

In addition to being a biomarker in cancers, increasing evidence has suggested that KRT18 is an important regulator in many diseases, including cancer. As keratin functions are related to cell injury and death. It was found that KRT18 expression level can be encouraged by EGR1, which then promotes cell apoptosis in non–small cell lung carcinoma (Zhang et al., 2014). And the diminished KRT18 expression was reported to improve the susceptibility of cytokine-induced death of cervical cancer cells (Sullivan et al., 2010) and inhibit cell migration and enhance paclitaxel sensitivity in lung cancer cells (Zhang et al., 2016). In breast cancer, KRT18 was reported to critically contribute to initiation of transforming growth factor β1 (TGF-β1)–induced epithelial–mesenchymal transition (EMT) in breast epithelial cells (Jung et al., 2016). Conversely, Shi et al. (2019) recently found that KRT18 knockdown increases BCRP (breast cancer resistant protein) expression and induces EMT process in human breast cancer MCF-7 cells (Shi et al., 2019). Moreover, a novel apoptotic function of KRT18 was discovered recently, which shows that KRT18 affects transcriptomes favoring apoptosis at both of transcriptional and alternative splicing (AS) levels in HeLa cells (Cheng et al., 2019). Transcriptional and posttranscriptional regulations are key molecular mechanisms contributing to the complex biological function of the human genome during gene expression process. It is worthy to investigate the regulation function of KRT18, a cytokeratin, at transcriptional and posttranscriptional levels in GC.

Alternative splicing regulation is related to transcripts of more than 95% of multiexon genes in human, and coordinated splicing networks regulate tissue and organ development (Pan et al., 2008; Baralle and Giudice, 2017). Deregulation of transcription and AS are all hallmarks of cancer (Bradner et al., 2017; Corbett, 2018). Studies on splice variants have found that incorrect splicing contributes to many human malignant tumorigenesis, including GC (da Cunha et al., 2016; Peng et al., 2019). The AS regulation of cancer-related genes allows the production of cancer-specific splicing isoforms, which are drivers of cancer progression or significant contributors to specific cancer hallmarks (El Marabti and Younis, 2018). Recently, emerging data have uncovered the cancer-specific AS isoforms of genes, which potentially were important targets for cancer therapy (Kahles et al., 2018). Meanwhile, the dynamic regulation of corresponding RNA splicing factors (SFs) and other regulators targeting important AS events (ASEs) play a critical role in tumorigenesis and serve as potential cancer treatment targets (Song et al., 2018). The previous study reported new regulatory function of KRT18, altering splicing events of apoptotic genes in HeLa cells (Cheng et al., 2019). But it remains unclear for the regulation mechanism of KRT18 on apoptosis process at transcriptional or posttranscriptional level in GC, in addition to undergoing caspase-mediated cleavage during epithelial apoptotic and necrotic cell death.

In the present study, we analyzed the expression level of KRT18 in 450 stomach adenocarcinoma (STAD) tissue samples available in TCGA database, showing a significant increase in cancer samples. We then explored the potential functions of KRT18 in GC AGS cells (human gastric adenocarcinoma cell line) using siRNA to knockdown KRT18 expression. The results showed that KRT18 selectively regulates expression of cell proliferation and apoptosis genes and affects the AS of pre-mRNAs from hundreds of genes, enriched in cancer-related pathways, which were validated by reverse transcription–quantitative polymerase chain reaction (RT-qPCR) approach. We then used the RNA-seq data of gastric tumor samples to further analyze the potential impact of KRT18 on AS regulation of cancer transcriptome. These results together support the conclusion that KRT18 extensively regulates diverse ASEs of genes enriched in cell cycle and apoptosis process, which was possibly by affecting SFs at transcriptional or posttranscriptional level, expanding the functional importance of keratins in apoptotic pathways at the AS level in GC.

## Materials and Methods

### Cell Culture and Transfections

Human GC cell line AGS was obtained from Procell (CL-0022, Wuhan, Hubei, China). The AGS cell line has been authenticated with STR analysis and tested for the free of mycoplasma contamination by the provider.

AGS cells were cultured with 5% CO_2_ at 37°C in Ham’s F-12 (Ham’s F-12 Nutrient Mixture), which were with 10% FBS (fetal bovine serum, 164210, Procell, Wuhan, Hubei, China), 100 U/mL penicillin, and 100 U/mL streptomycin (Hyclone). To silence the expression of KRT18 in AGS cells, we synthesized three siRNAs from Genepharma (Shanghai, China). The siRNA sequence against KRT18 mRNA sequence is shown in Table 1. According to the manufacturer’s protocol, three siRNAs were transfected into AGS cells using Lipofectamine 2000 (Invitrogen, Carlsbad, CA, United States), which were harvested after 48 h for following RT-qPCR analysis.

**TABLE 1:** The sequences of 3 siRNA against KRT18 mRNA sequence.

siRNA	Sense (5′-3′)	Antisense (5′-3′)
siRNA1	GGUUCCCGGAUCUCCGUGUTT	ACACGGAGAUCCGGGAACCTT
siRNA2	GCUGAUGACUUUAGAGUCATT	UGACUCUAAAGUCAUCAGCTT
siRNA3	GAGCUAGACAAGUACUGGUTT	ACCAGUACUUGUCUAGCUCTT

### Assessment of the Knockdown of *KRT18* by siRNA

We used housekeeping gene *GAPDH* (glyceraldehyde-3-phosphate dehydrogenase) as a control gene to assess whether *KRT18* was knocked down in AGS cells. Complementary DNA (cDNA) synthesis was performed according to standard procedures followed by 65°C for 5 min, 25°C for 10 min, and 42°C for 30 min using the Kit One-Step gDNA Removal and cDNA synthesis mix (AT311-02, Transgen Biotech, China) with PCR machine (Mycycler, Bio-Rad). And real-time quantification PCR was performed using HieffTM qPCR SYBR^®^ Green Master Mix (Low Rox Plus; YEASEN, Shanghai, China) to evaluate the knockdown inefficiencies of *KRT18* by siRNAs. The qPCR was run at 95°C for 5 min, followed by 40 cycles of 95°C for 10 s and 60°C for 30 s. Primer sequences used for RT-qPCR are presented in Table 2. The concentration of each transcript was then normalized to the *GAPDH* mRNA level using 2^–Δ^ ^Δ^ ^*CT*^ method (Livak and Schmittgen, 2001) to measure the transcript level of *KRT18*, with a non–reverse transcription group as a background.

**TABLE 2:** Primer sequences of KRT18 expression assessment used in RT-qPCR experiments.

KRT18-F	AAAGGCCTACAAGCCCAGAT
KRT18-R	CACTGTGGTGCTCTCCTCAA

### RNA Extraction and High-Throughput Sequencing

The AGS cells were ground into fine powder before RNA extraction. Total RNA was extracted with TRIZOL (Ambion), purified with two phenol–chloroform treatments treated with RQ1 DNase (Promega) to remove DNA. The quality and quantity of the purified RNA were determined by measuring the absorbance at 260 nm/280 nm (A260/A280) using smartspec plus (Bio-Rad) and using 1.5% agarose gel electrophoresis to verify RNA integrity. One microgram of total RNA for each sample was used for RNA-seq library preparation by KAPA Stranded mRNA-Seq Kit for Illumina^®^ Platforms (#KK8544). Polyadenylated mRNAs were purified by VAHTS mRNA capture beads (N401-01), fragmented, and converted into double-strand cDNA. Following end repair, Diluted Roche Adaptor (KK8726) was ligated to A tailing of the DNAs. After purification of ligation product and size fractioning to 300–500 bps, the ligated products were amplified and purified, quantified, and stored at −80°C before sequencing (Li et al., 2018). The strand marked with dUTP (the 2nd cDNA strand) is not amplified, allowing strand-specific sequencing. According to the manufacturer’s instructions, the libraries were applied to Illumina Novaseq 6000 system for 150-nt paired-end sequencing (ABLife Inc., Wuhan, China).

### RNA-seq Raw Data Clean and Alignment

Raw sequencing reads were first cleaned by discarding those containing more than 2-N bases, trimming off adaptors and low-quality bases using FASTX-Toolkit (version 0.0.13), and dropping the short reads less than 16 nt. These clean reads were subsequently aligned to the GRch38 genome by TopHat2 (Kim et al., 2013) with four mismatches. Uniquely mapped reads were ultimately used to calculate reads number and FPKM (paired-end fragments per kilobase of exon per million fragments mapped) value, mRNA expression level for each gene.

### Differentially Expressed Gene Analysis

The R Bioconductor package EdgeR (Robinson et al., 2010) was used to analyze the differentially expressed genes (DEGs) using RNA-seq data between the *KRT18*-KD and control groups. DEGs were determined based on the fold change (fold change ≥ 2 or ≤0.5) and *p*-value (*p* < 0.01).

Gene Ontology (GO) and enriched Kyoto Encyclopedia of Genes and Genomes (KEGG) pathway analyses were performed using KOBAS 2.0 server (Xie et al., 2011) to predict genes function and calculate the functional categories distribution frequency. Using hypergeometric test and Benjamini–Hochberg false discovery rate (FDR) controlling procedure, the enrichment of each pathway (corrected *p* < 0.05) was defined.

### Alternative Splicing Analysis

The ABLas pipeline as described previously (Xia et al., 2017) was used to define and quantify the ASEs between the *KRT18*-KD and control samples. In brief, detection of 10 types of canonical ASEs in each sample was based on the splice junction reads. These ASEs were exon skipping (ES), cassette exon (CE), alternative 5′ splice site (A5SS), alternative 3′ splice site (A3SS), mutual exclusive exon skipping (MXE), the MXE combined with alternative polyadenylation site (3pMXE), or with alternative 5′ promoter (5pMXE), intron retention (IR), A3SS&ES and A5SS&ES, and regulated ASEs (RASEs) (KRT18-affected ASAS events).

Alternative splicing ratios of all ASEs were calculated using the following formula: AS junction reads/(AS junction reads + M junction reads), where AS is altered splicing events, and M is model splicing events. We calculated and compared the changed AS ratio of alternatively spliced reads and constitutively spliced reads between *KRT18*-KD and control group samples, which was defined as the RASE ratio. After that, Student *t*-test was performed to evaluate the significance of the RASE ratio. The RASE ratio > 0.2 and *p* < 0.05 were set as the threshold for RASE detection.

### RT-qPCR Validation of DEGs and RASEs

To elucidate the validity of DEGs and RASEs in AGS cells, RT-qPCR was performed in this study for selected important DEGs and RASEs and normalized with the human housekeeping gene *GAPDH*. All primers for detecting genes transcript and specific splicing events are shown in Supplementary Table 6. To quantitatively analyze the two different splicing isoforms of a specific ASE using a qPCR approach, we designed two pairs of primers to specifically amplify each of these two isoforms after the initial synthesis of the first-strand cDNA using random primers. To achieve this specificity, we designed a primer pairing the splice junction of the constitutive exon and alternative exon (Supplementary Table 6). The RNA samples used for RT-qPCR were the same as that for RNA-seq. The RT-qPCR was respectively performed in triplicates for *KRT18*-KD and control samples.

### PI Staining to Detect Cell Cycle Distribution

Cultured cells with different treatments were resuspended in 95% ethyl alcohol at 4°C overnight and then washed twice with cold phosphate-buffered saline and treated with RNase for 30 min at room temperature followed by dying with PI in darkness at 4°C for 30 min using cell cycle detection kit (FXP0211-100, 4A Biotech Co. Ltd., Beijing, China) according to the manufacturer’s protocol. Next, cells were analyzed on a FACSAria flow cytometer (Cytoflex, Beckman). Red fluorescence intensity was detected, and DNA content was analyzed with ModFitWinTrial software.

### Cell Proliferation Assay

The proliferation capacity of the AGS cells was assessed with a CCK8 assay (HY-K0301, MCE), which was performed according to the manufacturer’s instructions. The absorbance was measured at 450 nm using an enzyme-linked immunosorbent assay plate reader (FC, Thermo). Each experiment was performed in triplicate at least.

### Flow Cytometric Analysis of Cell Apoptosis

One hundred five AGS cells were seeded in 24-well culture plates incubated for 24 h at 37°C and 5% CO_2_. Apoptosis was detected with flow cytometry (Cytoflex, Beckman) using double-staining with Fluor647-conjugated annexin V and PI (4A Biotech Co. Ltd., Beijing, China).

### Downloading RNA-seq Data of GC Samples

The RNA-seq data of GC samples were downloaded from TCGA database and GSE113255 (Kim et al., 2020) to analyze the expression of *KRT18* and regulation of AS in GC samples.

## Results

### *KRT18* Is Overexpressed in Stomach Adenocarcinoma Samples and Selectively Regulates the Expression of Genes Involved in Cell Proliferation and Apoptosis in AGS Cells

**FIGURE 1:**
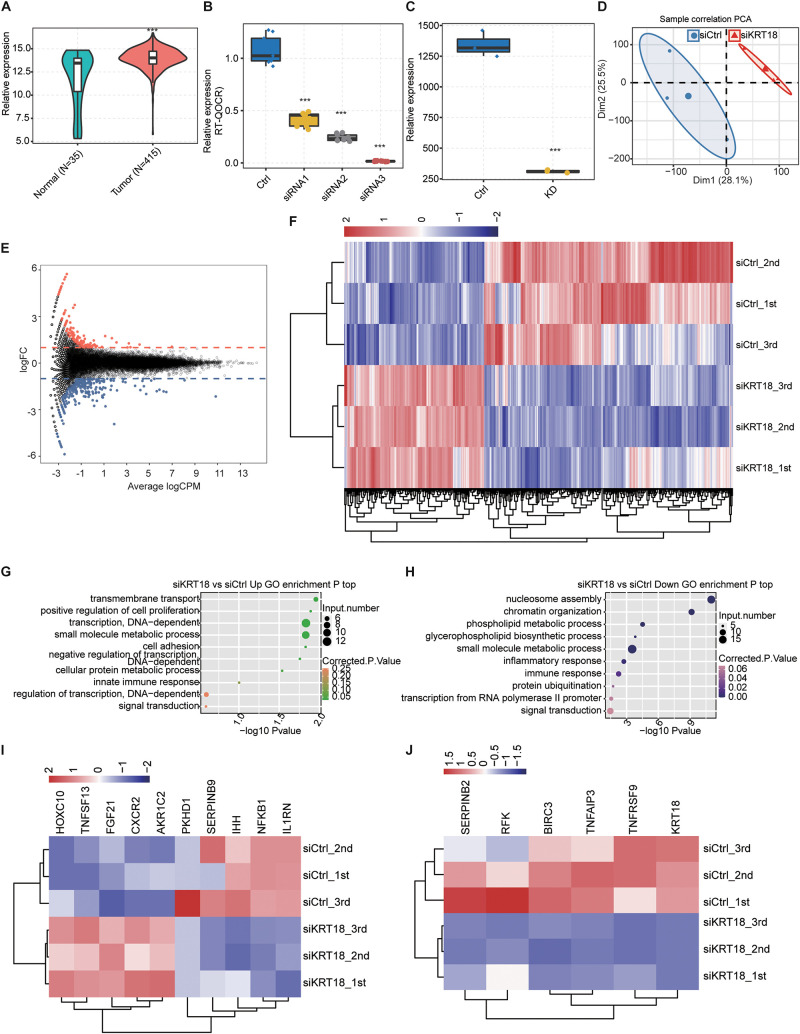
Overexpression of *KRT18* in stomach adenocarcinoma (STAD) and RNA-seq analysis of KRT18-regulated transcriptome profile in AGS cells. **(A)** Overexpression of *KRT18* in 450 stomach adenocarcinoma (STAD) samples from TCGA database, including 415 tumor tissue and 35 normal samples (para-carcinoma tissue). **(B)** *KRT18* expression quantified by qRT-PCR after knocking down *KRT18* in AGS cells using three siRNAs. **(C)** *KRT18* expression quantified by RNA sequencing data of AGS cells treated with *KRT18*-siRNA2, FPKM values were calculated as explained in section “Materials and Methods.” Error bars represent mean ± SEM. ***p < 0.001, Student *t*-test. **(D)** Principal **(F)** (PCA) of *KRT18*-KD versus control AGS cells based on FPKM value of all expressed genes. The samples were grouped by *KRT18*-KD and control, and the ellipse for each group is the confidence ellipse. **(E)** MA plot shows KRT18-regulated genes identified in AGS cells. Up-regulated genes are labeled in red, whereas down-regulated genes are labeled in blue. **(F)** Hierarchical clustering of DEGs in *KRT18*-KD and controls. FPKM values are log2-transformed and then median-centered by each gene. **(G,H)** The top 10 representative GO biological processes of up- and down-regulated genes. **(I)** Heatmap presenting deregulation expression of cell proliferation or apoptosis genes in *KRT18*-KD AGS cells. **(J)** Heatmap presenting repressed expression of the other six apoptotic genes in *KRT18*-KD AGS cells.

To investigate the connection between KRT18 and gastric carcinoma, we analyzed KRT18 expression pattern of STAD samples from The Cancer Genome Atlas (TCGA) database, including 415 tumor tissue and 35 normal (para-carcinoma tissue) samples. *KRT18* showed a significantly higher expression level in tumor tissues compared to normal tissues (Figure 1A). Among the staged 415 tumor tissue samples according to the standard of National Comprehensive Cancer Network stage, the expression of *KRT18* was significantly up-regulated in most tumor stages, more pronouncedly in late stages (Supplementary Figure 1A). We further performed survival analysis using the expression data but found no relationship between *KRT18* expression level and overall survival probability in the STAD samples from TCGA database (Supplementary Figure 1B).

In order to clearly understand the KRT18-affected gene expression regulation, we constructed *KRT18*-silenced AGS cell model with three biological replicates by siRNA and empty vector, followed with RNA-seq experiments and analysis. Using three synthetic siRNAs targeting mRNA of KRT18 (Table 1), we successfully knocked down *KRT18* in AGS cells and examined its expression by RT-qPCR (Figure 1B; primer details are shown in Table 2). The siRNA2-silenced *KRT18* AGS cells (*KRT18*-KD) were used in subsequent RNA-seq experiments.

Three biological replicating RNA-seq samples were constructed for *KRT18*-KD and control cells. After aligning quality filtered reads to the human GRCH38 genome and calculating the expression values in units of FPKM, the six samples totally yield 30,632 expressed genes (FPKM > 0), with 22,174–23,893 expressed genes in each sample (Supplementary Table 1). FPKM value of *KRT18* showed a significant decrease in *KRT18*-KD group compared to the control group (Figure 1C). Principal component analysis based on FPKM values of all expressed genes showed that knockdown of *KRT18* was the major factor globally influencing the gene expression pattern (Figure 1D).

Based on the RNA-seq data of above *KRT18*-KD and control cells, we used edgeR to further identify genes regulated by KRT18 at expression level. A total of 440 DEGs were identified between the *KRT18*-KD and control cells, including 153 up-regulated and 272 down-regulated genes (Supplementary Table 2), with criteria as fold change ≥ 1.5 or ≤0.67 and FDR < 0.05 (Figure 1E). The heatmap analysis of the expression patterns of the DEGs in RNA-seq samples showed a high consistency of the KRT18-mediated transcription in three biological replicates (Figure 1F). These results indicated that *KRT18*-KD significantly affected the gene expression level of a set of genes. To investigate the potential biological functions of these DEGs, we subjected all the DEGs to GO (Figure 1G) and KEGG enrichment analysis (Supplementary Figure 2). In the biological process terms of GO analysis, the up-regulated genes in the *KRT18*-KD group were mainly enriched in small molecule metabolic process, DNA-dependent transcription, and transmembrane transport, whereas down-regulated genes were mostly related to nucleosome assembly, chromatin organization, phospholipid metabolic process, and inflammatory/immune response (Figure 1H). The expression pattern of the cancer-related DEGs showed a high consistency of the KRT18-affected transcription in three biological replicate datasets (Figure 1I). Besides genes in Figure 1I, we also detected that six genes involved in apoptotic process were specifically down-regulated in si-KRT18 samples, including *BIRC3*, *SERPINB2*, *KRT18*, *RFK*, *TNFRSF9*, and *TNFAIP3* (Figure 1J). The results showed that KRT18 selectively affects genes enriched in DNA-dependent transcription and metabolic process pathways related to cancer development at transcriptional level.

### KRT18 Affects the Alternative Splicing Events of Genes Related to Cell Cycle and Apoptosis

**FIGURE 2:**
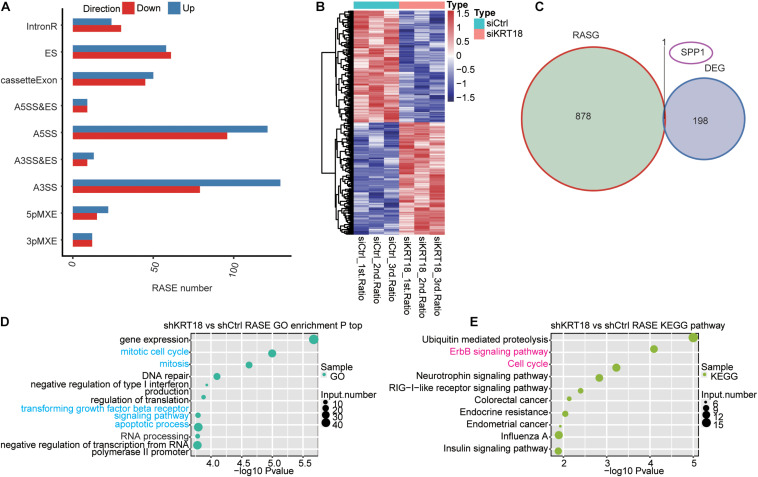
Analysis of KRT18-regulated alternative splicing in AGS cells. **(A)** Classification of KRT18-regulated alternatively spliced events in AGS cells. **(B)** Heatmap presenting AS ratio pattern of KRT18-affected ASEs. **(C)** Venn diagram shows the result of overlap analysis between KRT18-regulated differentially expressed genes (DEGs) and alternative splicing genes (RASGs). **(D)** The top 10 GO biological process terms and **(E)** KEGG functional pathway of the alternative splicing genes are shown in bubble plots.

Alternative splicing regulation is an important posttranscriptional regulation highly related to cancer (Chen and Weiss, 2015; Climente-Gonzalez et al., 2017). To uncover the role of KRT18 on AS, we further explored the KRT18-dependent ASEs in AGS cells using transcriptome sequencing data. A total of 20.6 ± 5.04 M junction reads, approximately 33.37–37.11% of all uniquely mapped reads, provided a solid foundation to explore AS changes (Supplementary Table 1). After comparing all junction reads of *KRT18*-KD and control AGS cells to the reference genome annotation, we detected 136,429 novel and 153,133 known splice junctions using TopHat2 (Supplementary Table 3). Using ABLas pipeline, we analyzed ASEs from the junction reads to identify 15,313 known ASEs (annotated ASEs) and 49,089 novel ASEs (unannotated ASEs) (ASE types are described in section “*Materials and Methods”*). We then identified 1,087 high-confidence RASEs by applying a stringent cutoff of *p* < 0.05, change AS ratio ≥ 0.2 (*KRT18*-KD vs. control group) (details can be found in Supplementary Table 4), indicating that KRT18 has a broad influence on AS of genes. RASEs mainly refer to alternative 3’ splice sites (A3SS), alternative 5’ splice sites (A5SS), ES, and CEs (Figure 2A). More extensively, the KRT18-affected ASE change patterns were highly consistent in three biological replicate datasets (Figure 2B). We analyzed the expression at transcriptional level of KRT18-affected alternative spliced genes (RASGs) and found that there are hardly any significant expression changes in RASGs (Figure 2C). By performing functional enrichment analysis, it was revealed that these RASGs were mainly enriched in gene expression, mitosis, cell cycle, DNA repair, negative regulation of type I interferon production, regulation of translation, TGF-β receptor signaling pathway, apoptotic process, and transcription, as well as RNA processing for GO biological process terms (Figure 2D; details can be found in Supplementary Table 5). Enriched KEGG pathways included those involved in ubiquitin-mediated proteolysis, erbB signaling pathway, cell cycle, neurotrophin signaling pathway, RIG-I–like receptor signaling pathway, colorectal cancer, endometrial cancer, influenza A, and insulin signaling pathway (Figure 2E; details can be found in Supplementary Table 5). These results indicated that KRT18-KD affected the AS of genes related to tumorigenesis pathways, including mitosis, cell cycle, and apoptosis.

### Validation of KRT18-Affected Cell Proliferation and Apoptosis Genes at Transcriptional and Alternative Splicing Level in AGS Cells

**FIGURE 3:**
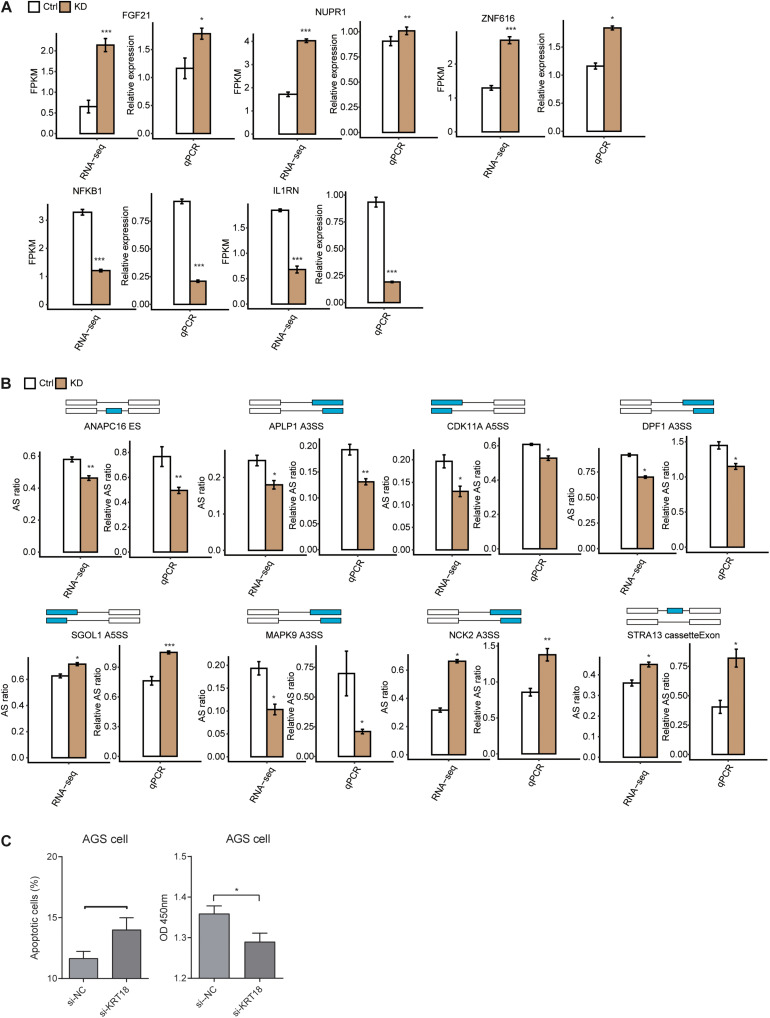
Validation of KRT18-affected expression and alternative splicing of genes related to cell proliferation and apoptosis in AGS cells. **(A)** Validation of KRT18-regulated gene expression by RT-qPCR in AGS cells. Gene expression quantified by RNA sequencing data and qRT-PCR. FPKM values were calculated as explained in section “Materials and Methods.” **(B)** Validation of KRT18-regulated alternative splicing events (RASEs) in genes related to cell cycle or apoptosis process by RT-qPCR in AGS cells. The schematic diagrams (top panel) depict the structures of ASEs, AS (altered splicing events), and M (model splicing events) (alternative exon was labeled in blue). The exon sequences are denoted by boxes and intron sequences by the horizontal line. RNA-seq quantification and RT-qPCR validation of ASEs are shown in the bottom panel. The altered ratios of AS events in RNA-seq were calculated using the formula: AS junction reads/(AS junction reads + M junction reads), whereas the altered ratios of AS events in RT-qPCR were calculated using the following formula: AS transcripts level/M transcripts level. Error bars represent mean ± SEM. *p < 0.05, **p < 0.01, ***p < 0.001, Student *t*-test. **(C)** Bar plot showing the apoptotic level (left panel) and proliferation level changes of AGS cells after KRT18-KD.

To focus on the role of KRT18 in regulating tumor occurrence and progression, we investigated the expression of DEGs enriched in cell proliferation and apoptosis and the AS of RASGs related to cell cycle and apoptosis (cancer-related) in *KRT18*-KD and control AGS cells, followed with verification experiments. RT-qPCR experiment quantifying DEG expression level showed that five of seven important DEGs, including *FGF21*, *NUPR1*, *ZNF616*, *NFKB1*, and *IL1RN*, were highly in agreement with the analysis results of RNA-seq data (Figure 3A and Supplementary Figure 3A). RT-qPCR experiment results showed that the AS ratios of 8 from 12 RASGs related to cell proliferation and apoptosis were highly in agreement with the transcriptome analysis results of RNA-seq data (Figure 3B and Supplementary Figure 3B). RT-qPCR experiment was performed using the primers designed to specifically amplify 7 validated DEGs and 12 ASEs in *KRT18*-KD and control AGS cells (Supplementary Table 6). Two important ASEs were located in *MAPK9* (*JNK2*) and *STRA13*, which have been well studied as key genes in cell proliferation in cancers.

To test whether KRT18 affects apoptosis and proliferation, we performed cellular apoptosis and proliferation experiments in GC cells, including AGS and MKN-45 cells. The KRT18-KD efficiencies were approximately 70 and 50% for AGS and MKN-45 cells, respectively. The results showed that KRT18-KD increased the apoptotic level and inhibited proliferation level in AGS cells (Figure 3C), whereas there was no difference for apoptotic and proliferation level in MKN-45 cell (Supplementary Figure 3C). As we detected mitosis and cell cycle pathways were enriched in KRT18-affected ASGs, we also checked the G2/G1 cell cycle transition ratio. However, we found there were no changes between *KRT18*-KD and control samples in both AGS and MKN-45 cells (Supplementary Figure 3D). These results demonstrated KRT18 could inhibit apoptosis and promote proliferation in gastric AGS cells.

### KRT18-Affected Alternative Spliced Genes Showed Similar Pattern in AGS Cells and KRT18-Dependent GC Samples

**FIGURE 4:**
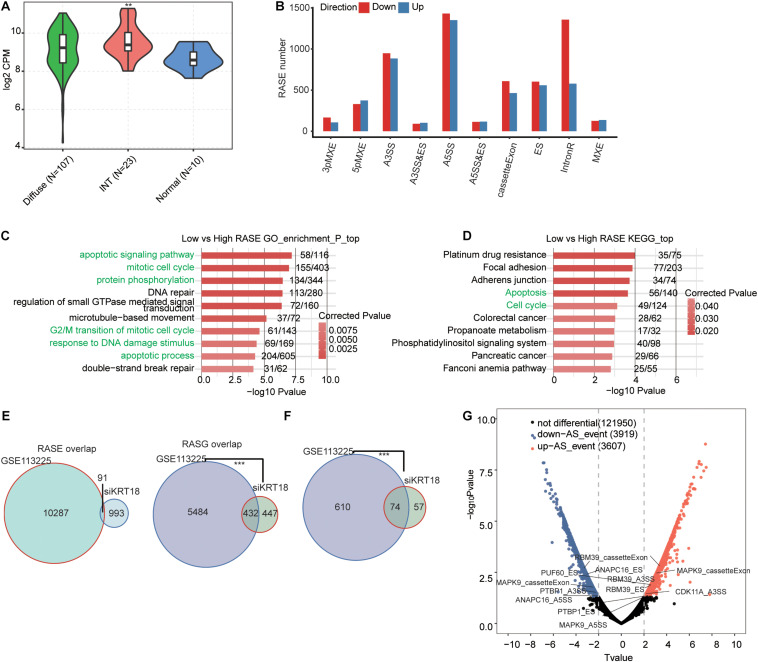
KRT18-regulated alternative spliced genes in AGS cells were similarly regulated in a KRT18-dependent manner in gastric cancer samples. **(A)** Violin plot of expression of KRT18 in 140 GC samples from GEO database (GSE113255), including 10 normal samples, 23 intestinal-type GC samples, and 107 diffuse-type GC samples. **(B)** Classification of deregulated alternative splicing events in gastric carcinoma samples with low expression of *KRT18* (*KRT18*-low group) compared to KRT18 highly expressed samples (*KRT18*-high group). **(C)** The top 10 GO biological processes terms and **(D)** KEGG functional pathways of deregulated alternative spliced genes between *KRT18*-low and -high gastric carcinoma samples. **(E)** Venn diagram shows the overlapping of all RASEs and RASGs in AGS cells and GC samples. **(F)** Venn diagram shows the overlapping of selected cancer-related RASGs in AGS cells and GC samples. ***p < 0.001, Hypergeometric test. **(G)** The volcano plots of RASGs in GC samples, differentially up-regulated ASEs (*T*-value ≥ 2, *p* < 0.01) are labeled red, whereas differentially down-regulated ones (*T* ≤ 0.5, *p* < 0.01) are labeled blue.

We then sought to study how the KRT18-affected ASEs also operate in GC samples. We downloaded a transcriptome dataset from GEO database (GSE113255) of 140 fresh-frozen tissues samples, including diffuse-type GC (*n* = 107), intestinal-type GC (*n* = 23), and normal gastric tissues (*n* = 10) (Kim et al., 2020). *KRT18* expression analysis results showed significantly higher expression in intestinal-type GC compared to normal samples (Figure 4A), consistent with the results in STAD samples from TCGA database.

To uncover the KRT18-associated ASEs in GC samples, we selected 50 GC samples, including 25 showing top high KRT18 expression (high group) and 25 showing bottom low (low group) from the 130 tumor tissue samples (Table 3). After aligning the filtered reads to human genome, an average of 9.7 ± 3 M junction reads per sample were identified (Supplementary Table 7). We then used the same pipeline to analyze ASEs and detected 37,120 known ASEs and 137,514 novel ASEs, among *KRT18* differently expressed GC samples. By comparing ASEs of the low group to the high group with the stringent cutoff of *p* ≤ 0.05, changed AS ratio ≥ 0.2, we identified 10,447 RASEs in 5,916 genes (RASGs) that were associated with KRT18 expression change in these 50 GC samples, mainly A5SS, A3SS, CE, ES, and IR events (Figure 4B). These data suggested that *KRT18* expression extensively affects ASEs in GC. RASGs were highly enriched (*p* ≤ 0.05) for apoptotic signaling pathway, mitotic cell cycle, protein phosphorylation, DNA repair, regulation of small GTPase-mediated signal transduction, microtubule-based movement, G2/M transition of mitotic cell cycle apoptotic process, response to DNA damage stimulus, and double-strand break repair in GO biological process term analysis (Figure 4C). Enriched KEGG pathways included platinum drug resistance, focal adhesion, apoptosis, cell cycle, colorectal cancer, phosphatidylinositol signaling system, and pancreatic cancer (Figure 4D; details can be found in Supplementary Table 8). The results indicate that the KRT18-associated RASGs play a role in gastric tumorigenesis in GC samples, in line with KRT18 regulation in AGS cells.

**TABLE 3:** The samples were grouped based on their KRT18 expression level regardless of tumor type.

Tumor type	Sample	Group description	KRT18 expression (CPM)
Diffuse	GSM3101199	KRT18_low	4.257
Diffuse	GSM3101104	KRT18_low	5.874
Diffuse	GSM3101115	KRT18_low	6.27
Diffuse	GSM3101196	KRT18_low	7.387
Diffuse	GSM3101081	KRT18_low	7.457
Diffuse	GSM3101192	KRT18_low	7.465
Diffuse	GSM3101177	KRT18_low	7.538
Diffuse	GSM3101153	KRT18_low	7.558
Diffuse	GSM3101158	KRT18_low	7.788
Diffuse	GSM3101137	KRT18_low	7.879
Diffuse	GSM3101145	KRT18_low	7.926
Diffuse	GSM3101194	KRT18_low	7.939
Diffuse	GSM3101111	KRT18_low	7.966
INT	GSM3101065	KRT18_low	8.014
Diffuse	GSM3101093	KRT18_low	8.048
Diffuse	GSM3101178	KRT18_low	8.053
Diffuse	GSM3101092	KRT18_low	8.07
Diffuse	GSM3101200	KRT18_low	8.12
Diffuse	GSM3101186	KRT18_low	8.132
Diffuse	GSM3101139	KRT18_low	8.164
INT	GSM3101069	KRT18_low	8.175
Diffuse	GSM3101067	KRT18_low	8.289
Diffuse	GSM3101148	KRT18_low	8.335
Diffuse	GSM3101195	KRT18_low	8.35
Diffuse	GSM3101112	KRT18_low	8.362
INT	GSM3101107	KRT18_high	11.292
INT	GSM3101130	KRT18_high	11.053
Diffuse	GSM3101180	KRT18_high	11.046
INT	GSM3101122	KRT18_high	11.042
Diffuse	GSM3101127	KRT18_high	10.857
Diffuse	GSM3101185	KRT18_high	10.752
Diffuse	GSM3101198	KRT18_high	10.751
Diffuse	GSM3101087	KRT18_high	10.694
INT	GSM3101083	KRT18_high	10.66
Diffuse	GSM3101075	KRT18_high	10.629
Diffuse	GSM3101100	KRT18_high	10.564
Diffuse	GSM3101156	KRT18_high	10.533
Diffuse	GSM3101099	KRT18_high	10.436
Diffuse	GSM3101108	KRT18_high	10.42
Diffuse	GSM3101109	KRT18_high	10.392
Diffuse	GSM3101064	KRT18_high	10.368
Diffuse	GSM3101098	KRT18_high	10.351
Diffuse	GSM3101146	KRT18_high	10.296
Diffuse	GSM3101181	KRT18_high	10.249
Diffuse	GSM3101172	KRT18_high	10.231
Diffuse	GSM3101183	KRT18_high	10.207
Diffuse	GSM3101188	KRT18_high	10.196
INT	GSM3101138	KRT18_high	10.173
Diffuse	GSM3101136	KRT18_high	10.17
Diffuse	GSM3101169	KRT18_high	10.146

Considering that tumor tissue is complicated in cell type and deregulated genes, these KRT18-dependent RASEs could be regulated by other factors. Then, we compared these RASEs and RASGs to those KRT18-affected in AGS cells and found that RASGs have a significant cooperation between gastric tissue samples and AGS cells, while it is not for RASEs (Figure 4E). This is also present in the RASGs enriched in cell proliferation, cell cycle, and apoptosis in GO biological process terms (Figure 4F). It is indicated that KRT18 may regulate similar genes, especially cell proliferation and apoptosis related, at the AS level in GC samples and cell lines, but not limited in the same splicing events. Besides, the validated RASGs in AGS cells were also significantly alternatively spliced in *KRT18*-high vs. *KRT18*-low GC tissue samples (Figure 4G).

### KRT18 Affects Splicing Factors at Posttranscriptional Level in AGS Cells

**FIGURE 5:**
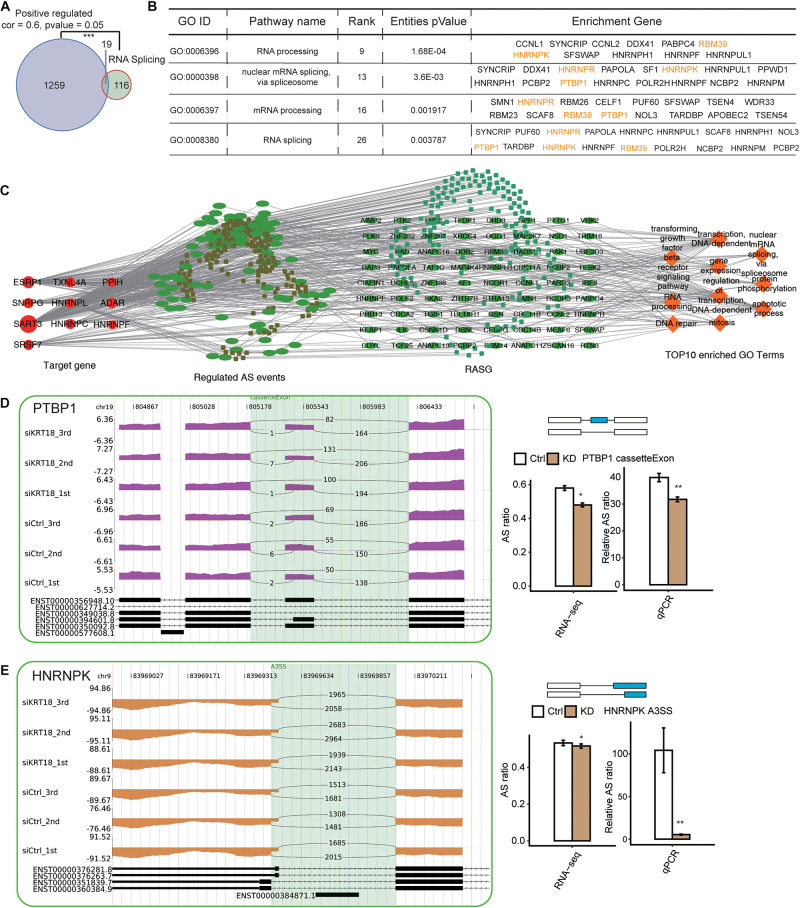
Deregulation of splicing factors by KRT18 in GC samples and AGS cells. **(A)** Venn diagram shows the overlapping between KRT18-coexpressed genes positively and genes in RNA splicing–related terms from GO database. ***p < 0.001, hypergeometric test. **(B)** RNA splicing–related terms in GO biological process analysis of the RASGs from RNA-seq data in AGS cells. Validated RASGs using RT-qPCR in AGS cells were labeled in yellow. **(C)** The coderegulation of alternative splicing network between top10 hub RBPs (the leftmost part and the size of the circle represented the number of connections) and RASEs (the middle left part, which includes only NIR RASEs). The top enriched GO biological process of codisturbed RASGs (the middle right part) was shown in orange (the rightmost part). **(D,E)** Validation of two RASEs, cassette exon in *PTBP1* **(C)**, and A3SS in *HNRNPK* **(D)**, by RT-qPCR in AGS cells. IGV sashimi plots show ASE changes occurred in *KRT18*-KD cells and control (left panel), and the transcripts for the gene are shown below (right panel, top). The schematic diagrams (right panel, top) depict the structures of ASEs, AS (altered splicing events), and M (model splicing events) (alternative exon was labeled in blue). The exon sequences are denoted by boxes and intron sequences by the horizontal line. RNA-seq quantification and RT-qPCR validation of ASEs are shown in the bottom of right panel. The altered ratios of AS events in RNA-seq were calculated using the following formula: AS junction reads/(AS junction reads + M junction reads), whereas the altered ratios of AS events in RT-qPCR were calculated using the formula: AS transcripts level/M transcripts level. Error bars represent mean ± SEM. *p < 0.05, **p < 0.01, Student *t*-test.

With the purpose of preliminarily inquire the potential AS regulation mechanism of KRT18, a non-canonical RNA-binding protein, we analyzed the relationship between KRT18-mediated transcription changes and ASEs and SFs in RNA-seq data of AGS cells and GC tissue samples. By coexpression analysis, we identified 1,278 KRT18-coexpressed genes (correlation coefficient ≥ 0.6, *p* < 0.05) in GC samples. We found the 1,278 coexpressed genes had a significant overlap with RNA splicing–related genes (19 overlapped genes), suggesting the relationship between KRT18 and RNA splicing regulators at transcriptional level (Figure 5A). As for the posttranscriptional regulation related to *KRT18*, the RASGs in *KRT18*-high vs. 25 *KRT18*-low GC tissue samples also enriched in RNA splicing term (details can be found in Supplementary Table 8). These results indicated that KRT18 affects gene ASEs possibly by modulating the expression or AS of some SFs. We then constructed a network between KRT18-coexpressed SFs and affected ASEs by calculating the correlation coefficient between them. A total of 463 SF-ASE pairs were obtained (correlation > 0.7 and *p* < 0.01), including 10 SFs and 272 ASEs, suggesting the substantial relationship between SFs and ASEs. We then analyzed the enriched functions of genes from the 272 ASEs. The top 10 enriched GO BP terms showed specific enrichment in apoptotic process, RNA splicing, transcription, and mitosis (Figure 5C).

Moreover, we found that KRT18-affected RASGs were significantly enriched in RNA processing and splicing (Figure 4B). Using RT-qPCR experiment, we verified the AS changes of four SFs (Figures 5D,E and Supplementary Figure 4), including *PTBP1*, *HNRNPK*, *HNRNPR*, and *RBM39*, identified from RNA-seq data in *KRT18*-KD and control AGS cells (primer details are shown in Supplementary Table 6). CE in *PTBP1* (Figure 5D) and A3SS in *HNRNPK* (Figure 5E) were verified with significant change in *KRT18*-KD vs. control group, in agreement with the transcriptome analysis results of RNA-seq data. These results suggested that KRT18 could affect global AS profile by modulating expression and AS of key SFs.

## Discussion

KRT18 is a cytokeratin in epithelial and endothelial cells reported to be aberrantly expressed and regarded as a biomarker and important regulator in many cancers, including GC (Yin et al., 2016; Zhang et al., 2016, 2019a; Bozza et al., 2018; Nagel et al., 2018; Golob-Schwarzl et al., 2019). In addition to be a biomarker in cancers, increasing evidences have suggested that KRT18 is an important regulator in many diseases, including cancer. It can impress cell migration, enhance drug sensitivity, and affect EMT process via signaling pathways in breast cancer or non–small cell lung carcinoma, as well as regulate FAS-mediated apoptotic pathway at transcriptional and AS levels in HeLa cells (Zhang et al., 2014, 2016; Jung et al., 2016; Cheng et al., 2019; Shi et al., 2019). In this study, we found KRT18-KD could inhibit cell proliferation and promote apoptosis in AGS cells. Nevertheless, it remains unclear for the regulation mechanism of cytokeratin proteins such as KRT18 on apoptosis process at transcriptional or posttranscriptional level in GC. In the present study, we performed experiments and bioinformatics analysis to identify what role KRT18 plays in GC and explore the potential regulatory mechanism.

We analyzed the expression level of *KRT18* in 450 STAD tissue samples from TCGA database and 140 GC samples from GEO database and found a significant overexpression in gastric tumor samples (Figures 1A, 4A), consistent with the biomarker function of KRT18 in GC (Bilici, 2015; Nagel et al., 2018). We then explored the potential functions of KRT18 in GC AGS cells by silencing *KRT18* with siRNA and unbiased RNA-seq analysis. Notably, KRT18 selectively affects the expression of genes enriched in cell proliferation, DNA-dependent transcription, and apoptotic process (Figures 1G,H), catering to the role of KRT18 in affecting EMT process and regulating apoptotic genes at transcriptional level (Jung et al., 2016; Cheng et al., 2019; Pastushenko and Blanpain, 2019). We found KRT18-KD promoted apoptosis and inhibited cell proliferation in AGS cells, but not in MKN-45 cells, which may be attributed to the insufficient knockdown efficiency in MKN-45 cells. We found that KRT18 up-regulated the expression of NFKB1 and IL1RN, whereas the FGF21, NUPR1, and ZNF616 were negatively regulated in AGS cells. KRT18-affected FGF21 was reported to activate the FGF signaling pathway by binding FGFR and have therapeutic potential in GC as a diagnostic and prognostic biomarker (Degirolamo et al., 2016; Zhang et al., 2019b). The transcription factor, NUPR1, can convert stress signals into a program of expression and participate in regulating cell cycle, apoptosis, autophagy, and DNA repair response processes (Chowdhury et al., 2009; Santofimia-Castano et al., 2018). It has been reported that NFKB1 was one of the five subunits of nuclear factor κB (NF-κB), which is widely implicated in carcinogenesis (Concetti and Wilson, 2018), and IL1RN was an angiogenesis inhibitor in GC (Gong et al., 2018). Further research can be carried out to study the regulatory mechanism of KRT18 on these genes.

In addition to expression regulation, bioinformatics analysis using RNA-seq data showed that KRT18 also modulates AS of many genes, which were enriched in mitosis, cell cycle, DNA repair, regulation of translation, TGF-β receptor signaling pathway, apoptotic process, transcription, and ERBB signaling pathway (Figures 2D,E). These functional pathways mostly reappeared in the analysis results of GC tissue samples (Figures 4C,D). The KRT18-affected RASGs in AGS cells and gastric samples were significantly overlapped, especially cell cycle and apoptosis genes, but not related to the same ASEs (Figures 4F,G), indicating that the effect of KRT18 on AS might be closely related to the development of GC. It was known that AS is tightly linked to tumors, which can produce different proteins with different structures and functions associated with carcinogenesis (Zhao et al., 2015; Sveen et al., 2016). Many studies have found that GC-related genes are differentially spliced (Li and Yuan, 2017), suggesting the important implication of AS in GC development.

Here we noted that validated ASEs regulated by KRT18 mostly located in genes related to mitosis, cell cycle, apoptotic process, and ERBB signaling pathway, including *ANAPC16* (anaphase promoting complex subunit 16), *APLP1* (amyloid beta precursor like protein 1), *CDK11A* (cyclin-dependent kinase 11A), *DPF1* (double PHD fingers 1), *SGOL1* (shugoshin 1), *MAPK9* (mitogen-activated protein kinase 9), *NCK2* (NCK adaptor protein 2), and *STRA13* (stimulated by retinoic acid gene 13 protein, also known as DEC1). These AS genes were also validated in GC samples with differential KRT18 (Figure 4G). The occurrence and progression of cancers, including GC, are necessarily accompanied by the dysregulation of mitotic cell cycle and apoptotic process (Bonomi et al., 2013; Cress et al., 2017). KRT18-affected NCK2 and JNK2 at the AS level are related to ERBB/HER/EGFR signaling pathway. This signaling pathway activation is correlated with tumorigenesis, cancer metastasis, prognosis, and overall survival in many cancers (Wang, 2017). Especially, the amplification, overexpression, and various splices of *ERBB2* are independent prognostic factors in GC (Volpi et al., 2019). NCK2 can modulate cell motility by interacting with focal adhesion through its SH3/SH2 domain (Goicoechea et al., 2002) and may influence tumor aggressiveness by mediating cell–extracellular matrix interactions in ovarian cancer (Fanelli et al., 2018) and promote melanoma progression *in vitro* and *in vivo* (Labelle-Cote et al., 2011). *MAPK9* encodes a serine/threonine-protein kinase (also named JNK2), distributed in tissue broadly. The functions of JNK2 in regulation of cancer cell apoptosis and survival have been highlighted in previous study (Wu et al., 2019). Crosstalk between JNK and other pathways, such as NF-κB, p38, and their sharing common upstream activators, is critical for cancer programming and may act synergistically to regulate cancer cell survival (Svensson et al., 2011; Ruan et al., 2015). JNK2 has been reported to be a mediator of cell apoptosis and death (Arbour et al., 2002; Zhong et al., 2007) and also induce cell survival in the contrary (Hochedlinger et al., 2002; Liu et al., 2004), underlining that the functional mechanism of JNK2 in GC needs to be further investigated. So far, there are at least four known JNK2 transcript variants, related to the alternate selection between two middle exons and the alternate splicing at the C terminus, which show different substrate-binding and self- or substrate-phosphorylation activities and expression level (Kallunki et al., 1994; Dreskin et al., 2001). Moreover, the novel JNK2 transcript variants were shown to have different stimulation activities on AP-1, a regulator of cell proliferation and survival (Wang et al., 2010). This result indicates that KRT18 could contribute to tumorigenesis via regulating the AS of *JNK2* or *NCK2*.

In addition, we found the significant coexpression between KRT18 and SFs in GC samples and KRT18-affected AS of SFs in AGS cells and GC samples (Figure 5 and Supplementary Table 8). By constructing the network between SFs and ASEs, we found 10 SFs were tightly related to the KRT18-affected ASEs. These interacted ASE genes were also enriched in apoptosis functional terms (Figure 5C), suggesting that the dysregulation of SFs at transcriptional or posttranscriptional levels may contribute to the result that KRT18 extensively regulates gene AS. The SF alternations, including somatic mutation, transcriptional alteration, and their functional impactions in human tumor development, have been extensively studied (Anczukow and Krainer, 2016; Rahman et al., 2020). Here PTBP1 and HNRNPK splicing patterns were both validated to be KRT18-dependent in AGS cells and GC samples. PTBP1 was originally identified as an SF and a promoter in multiple cancers by regulating oncogenes or tumor suppressors, including PKM2, MRP1, and FGFR1 (Wang et al., 2017; Georgilis et al., 2018). Similarly, HNRNPK has been reported to be multifunctional protein overexpressed in several human cancers, regulating both oncogenic and tumor-suppressive pathways through a bevy of chromatin-, DNA-, RNA-, and protein-mediated activation (Gallardo et al., 2016), such as inhibiting tumor growth through p53/p21/CCND1 axis *in vivo* (Huang et al., 2017), and promotes gastric tumorigenesis through regulating CD44E AS (Peng et al., 2019). Thus, KRT18 may regulate the expression and AS of these SFs to regulate many ASEs.

In this study, we have successfully demonstrated the KRT18 impaction on gene expression and AS in AGS cells and GC samples, possibly via affecting some key SFs. We, for the first time, constructed the interaction network between KRT18-coexpressed SFs and KRT18-affected ASEs, providing a novel mechanism how KRT18 affects AS in cells. The results that KRT18 affects proliferation and apoptotic genes, which is critical in tumorigenesis and cancer progression at transcriptional or posttranscriptional level, underline that the well-known caspase-cleaved cytokeratin KRT18 might conversely regulate proliferation and apoptotic pathways, which expands the functional importance of keratins in GC. Further study of KRT18-affected AS should contribute to a precise understanding of keratins directing tumorigenesis and potentially KRT18-targeted therapies.

## Data Availability Statement

RNA-seq data in this publication have been deposited in NCBI’s Gene Expression Omnibus and are accessible through GEO series accession number GSE158242. The clinical samples data can be obtained from The Cancer Genome Atlas (TCGA) database and GEO database (GSE113225).

## Author Contributions

W-GD and BC contributed to the study design. BC, XX, D-DL, and Y-TX prepared the experiments and data analysis. W-GD and BC wrote the manuscript. All authors read and approved the final manuscript.

## Conflict of Interest

The authors declare that the research was conducted in the absence of any commercial or financial relationships that could be construed as a potential conflict of interest.

## Supplementary Material

Supplementary Figure 1 Analysis of KRT18 expression levels and prognostic in stomach adenocarcinoma (STAD) samples from TCGA database. **(A)** Distribution of expression levels of *KRT18* in normal samples and different stages of STAD samples. Statistical analysis was performed by Student *t*-test: ^∗∗^*p* < 0.01, ^∗∗∗^*p* < 0.001. Stomach adenocarcinoma was staged according to standard of National Comprehensive Cancer Network (NCCN) stage. **(B)** Overall survival (OS) in STAD patients with top 50% versus bottom 50% of KRT18 expression.

Supplementary Figure 2 The top 10 representative KEGG pathways of up- **(A)** and down-regulated genes **(B)**.

Supplementary Figure 3 Validation of DEGs **(A)** and RASEs **(B)** of genes related to cell cycle or apoptosis process by RT-qPCR in AGS cells. **(A)** Gene expression quantified by RNA sequencing data and qRT-PCR. FPKM values were calculated as explained in section “Materials and Methods.” **(B)** The schematic diagrams (top panel) depict the structures of ASEs, AS (altered splicing events), and M (model splicing events) (alternative exon was labeled in blue). The exon sequences are denoted by boxes and intron sequences by the horizontal line. RNA-seq quantification and RT-qPCR validation of ASEs are shown in the bottom panel. The altered ratio of AS events in RNA-seq were calculated using the formula: AS junction reads/(AS junction reads + M junction reads), whereas the altered ratios of AS events in RT-qPCR were calculated using the formula: AS transcripts level/M transcripts level. Error bars represent mean ± SEM. ^∗^*p* < 0.05, ^∗∗^*p* < 0.01, ^∗∗∗^*p* < 0.001, Student *t*-test. **(C)** Bar plot showing the apoptotic level (left panel) and proliferation level changes of MKN-45 cells after KRT18-KD. **(D)** Bar plot showing the G2/G1 ratio in KRT18-KD and control samples by cell cycle experiment. AGS cells (left panel) and MKN-45 cells (right panel) were shown.

Supplementary Figure 4 Validation of RASEs in splicing factor genes, HNRNPR **(A)** and RBM39 **(B)**, by RT-qPCR in AGS cells. IGV sashimi plots show ASE changes occurred in *KRT18*-KD cells and control (left panel), and the transcripts for the gene are shown below (right panel, top). The schematic diagrams (right panel, top) depict the structures of ASEs, AS (altered splicing events), and M (model splicing events) (alternative exon was labeled in blue). The exon sequences are denoted by boxes and intron sequences by the horizontal line. RNA-seq quantification and RT-qPCR validation of ASEs are shown in the bottom of right panel. Error bars represent mean ± SEM. ^∗^*p* < 0.05, ^∗∗∗^*p* < 0.001, Student *t*-test. The altered ratios of AS events in RNA-seq were calculated using the formula: AS junction reads/(AS junction reads + M junction reads), whereas the altered ratios of AS events in RT-qPCR were calculated using the formula: AS transcripts level/M transcripts level.

## Abbreviations

KRT18: keratin 18
DEGs: differential expressed genes
AS: alternative splicing
ASEs: alternative splicing events
RASEs: KRT18-affected alternative splicing events
RASGs: KRT18-affected alternative spliced genes
SFs: splicing factors
TCGA: The Cancer Genome Atlas.
