# Supplementary material for: KRT18 Modulates Alternative Splicing of Genes Involved in Proliferation and Apoptosis Processes in Both Gastric Cancer Cells and Clinical Samples
Source: Front Genet. 2021 Jul 5;12:635429. doi: 10.3389/fgene.2021.635429 (PMC8287183; doi:10.3389/fgene.2021.635429)

Supplementary Material

Supplementary figure 1 Analysis of KRT18 expression levels and prognostic in stomach adenocarcinoma (STAD) samples from TCGA database.

(A) Distribution of expression levels of KRT18 in normal samples and different stages of STAD samples. Statistical analysis was performed by Student's t-test: **p < 0.01, ***p < 0.001. Stomach adenocarcinoma was staged according to standard of National Comprehensive Cancer Network (NCCN) stage. (B) Overall survival (OS) in STAD patients with top 50% versus bottom 50% of KRT18 expression.

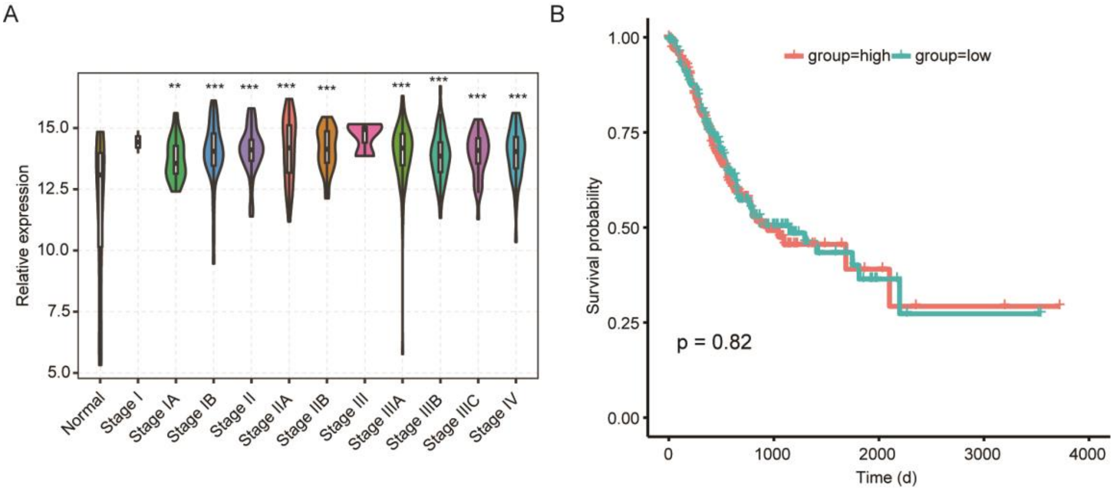

Supplementary figure 2 The top 10 representative KEGG pathways of up- (A) and down-regulated genes (B).

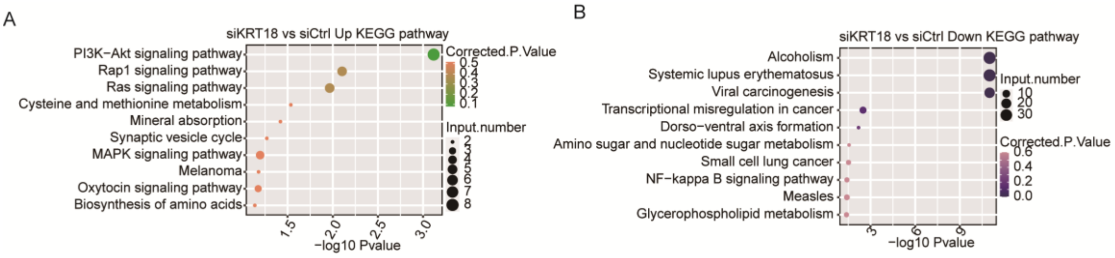

Supplementary figure 3 Validation of DEGs (A) and RASEs (B) of genes related to cell cycle or apoptosis process by RT-qPCR in AGS cells.

(A) Gene expression quantified by RNA sequencing data and qRT-PCR. FPKM values were calculated as that has been explained in Materials and Methods. (B) The schematic diagrams (top panel) depict the structures of ASEs, AS (altered splicing events) and M (model splicing events) (alternative exon was label in blue). The exon sequences are denoted by boxes and intron sequences by the horizontal line. RNA-seq quantification and RT-qPCR validation of ASEs are shown in the bottom panel. The altered ratio of AS events in RNA-seq were calculated using the formula: AS junction reads / (AS junction reads + M junction reads); while the altered ratio of AS events in RT-qPCR were calculated using the formula: AS transcripts level / M transcripts level. Error bars represent mean ±SEM. *$p < 0.05$, **$p < 0.01$, ***$p < 0.001$, Student's t-test. (C) Bar plot showing the apoptotic level (left panel) and proliferation level changes of MKN-45 cells after KRT18-KD. (D) Bar plot showing the G2/G1 ratio in KRT18-KD and control samples by cell cycle experiment. AGS cells (left panel) and MKN-45 cells (right panel) were shown.

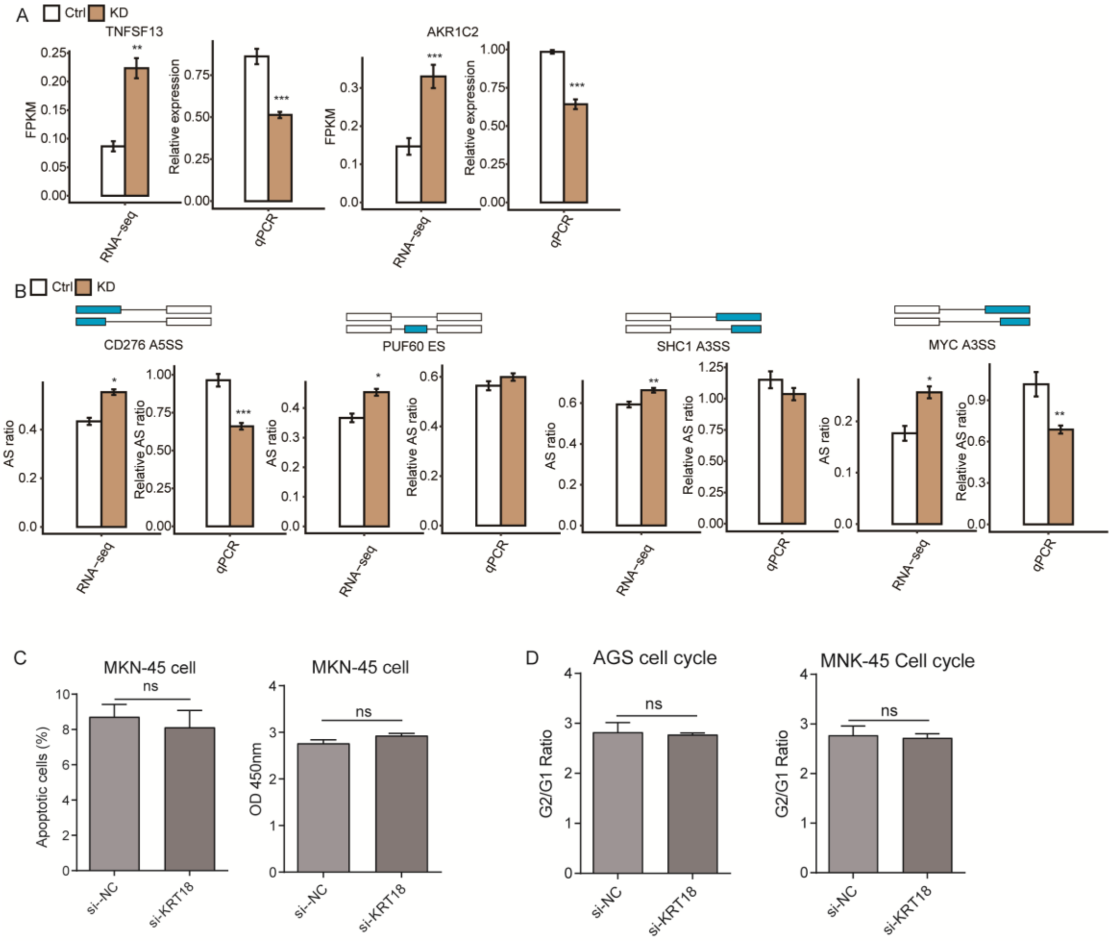

Supplementary figure 4 Validation of RASEs in splicing factor genes, HNRNPR (A) and RBM39 (B), by RT-qPCR in AGS cells.

IGV sashimi plots show ASE changes occurred in KRT18-KD cells and control (Left panel) and the transcripts for the gene are shown below. (Right panel, top). The schematic diagrams (Right panel, top) depict the structures of ASEs, AS (altered splicing events) and M (model splicing events) (alternative exon was label in blue). The exon sequences are denoted by boxes and intron sequences by the horizontal line. RNA- seq quantification and RT-qPCR validation of ASEs are shown in the bottom of right panel. Error bars represent mean ±SEM. *p < 0.05, ***p < 0.001, Student’s t-test. The altered ratio of AS events in RNA-seq were calculated using the formula: AS junction reads / (AS junction reads + M junction reads); while the altered ratio of AS events in RT-qPCR were calculated using the formula: AS transcripts level / M transcripts level.

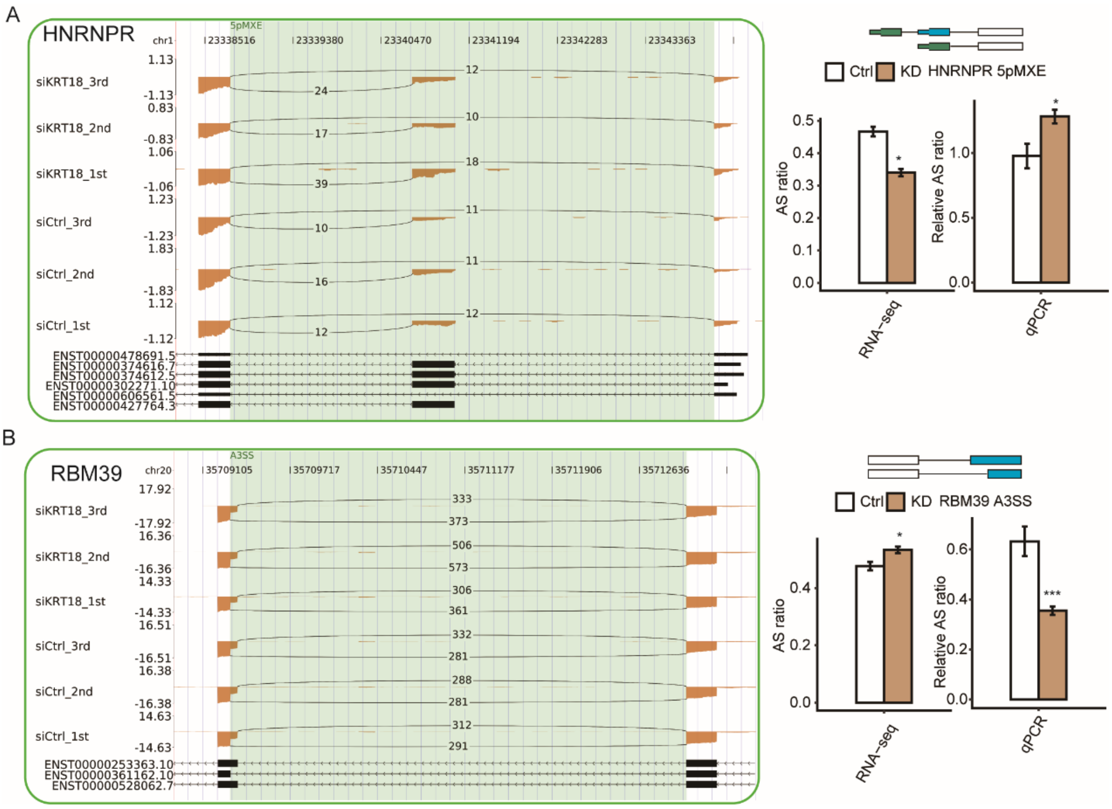

Supplement: Supplementary Figure 1 — Analysis of KRT18 expression levels and prognostic in stomach adenocarcinoma (STAD) samples from TCGA database. (A) Distribution of expression levels of KRT18 in normal samples and different stages of STAD samples. Statistical analysis was performed by Student t-test: ∗∗p < 0.01, ∗∗∗p < 0.001. Stomach adenocarcinoma was staged according to standard of National Comprehensive Cancer Network (NCCN) stage. (B) Overall survival (OS) in STAD patients with top 50% versus bottom 50% of KRT18 expression. [file Data_Sheet_1.PDF]
